# Monthly Report of Deaths in Buffalo

**Published:** 1851-08

**Authors:** W. L. G. Smith

**Affiliations:** Clerk


### *Monthly Report of Deaths in Buffalo.*

BOARD OF HEALTH, *Buffalo*, July, 1851.

Number of deaths reported to the Board for the month of June last:—Males, 47; females, 21—total, 68.

Age—Adults, 31; children, 37. Nation—Americans, 17; Germans, 28; Irish, 15; English, 2; Scotch, 4; French, 1; Norwegian, 1. Diseases—Consumption, 11; summer complaint, 7; old age, 2; casualties, 3; pleurisy, 1; jaundice, 1; gastritis, 1; delirium tremens, 2; liver complaint, 1; drowned, 3; poison, 1; unknown, 19; asphyxia, 1; fever, 5; scarlet fever, 1; pneumonia, 1; small pox, 2; inflammation on the brain, 1; whooping cough, 1; hydrocephalus, 1; convulsion, 1; dentition, 1; croup, 1.

By order of the Board,

W. L. G. SMITH, Clerk.

